# *Cystoisospora belli* Gallbladder Infection in a Liver Transplant Donor

**DOI:** 10.1155/2018/3170238

**Published:** 2018-07-02

**Authors:** Clifford Akateh, Christina A. Arnold, Dathe Benissan-Messan, Anthony Michaels, Sylvester M. Black

**Affiliations:** ^1^General and Gastrointestinal Surgery, Department of Surgery, The Ohio State University Wexner Medical Center, Columbus, OH 43210, USA; ^2^Department of Pathology, The Ohio State University Wexner Medical Center, Columbus, OH 43210, USA; ^3^Division of Gastroenterology, Hepatology and Nutrition, Department of Internal Medicine, The Ohio State University Wexner Medical Center, Columbus, OH 43210, USA; ^4^Division of Transplant Surgery, Department of Surgery, The Ohio State University Wexner Medical Center, Columbus, OH 43210, USA

## Abstract

**Introduction:**

*Cystoisospora belli* (previously *Isospora belli*) is a parasitic protozoan of the human gastrointestinal system. It rarely causes symptoms in immunocompetent hosts but can cause severe diarrhea in immunocompromised patients, with a rate of recurrence and risk of dissemination. Gallbladder infections are however rare. The treatment of choice for symptomatic patients is a 7–10-day course of trimethoprim-sulfamethoxazole.

**Case:**

In this case, we report on an incidental finding of *Cystoisospora belli* organisms in the donor gallbladder following a transplant cholecystectomy. There was no report of symptoms in the donor. The recipient was treated with a course of trimethoprim-sulfamethoxazole, without evidence of cystoisosporiasis. Given the risk of recurrence in immunocompromised hosts, the patient will continue to be monitored for reactivation in the future.

**Conclusion:**

Despite advances in transplant protocols and screening, disease transmission from the donor to recipient still occurs in about 0.2% of all organ transplants. With the increased use of organs from drug overdose victims and other high-risk donors, practitioners (including pathologists, hepatologists, and surgeons) must maintain a high index of suspicion for such potentially harmful organisms.

## 1. Introduction

*Cystoisospora belli*, previously called *Isospora belli* (*C. belli*), is an intracellular protozoan of the intestinal epithelium. Although present worldwide, it is a less common cause of protozoal diarrhea, compared to *Toxoplasma* and *Cryptosporidium*. It is most prevalent in the tropical regions [1–3]. In the West, *Cystoisospora belli* is associated with HIV/AIDS infection and occasionally diarrhea in travelers [4–8]. It is transmitted by the ingestion of contaminated produce or water [3]. The oocysts can be seen on light microscopy with hematoxylin and eosin stain of tissue samples. Modified acid-fast stains can also be used in challenging ova in stool samples [9]. In addition, PCR assays to detect the organism in stool samples exist [10, 11].

In immunocompetent individuals, the protozoan occasionally causes mild episodes of watery diarrhea, fever, nausea, vomiting, and malabsorption, but overall is typically asymptomatic [12]. On the other hand, immunocompromised patients experience more severe and prolonged symptoms [13]. In 1994, Benator et al., reported on the first case of *C. belli*-induced acalculous cholecystitis [14]. Extraintestinal manifestations have also been reported in these patients as well, including gallbladder and biliary tract infections [13–17]. In recent years, there have been case reports of *C. belli* infections in immunocompetent individuals. Most of these cases involve a recent immigrant from the subtropics [5, 7, 8]. Surprisingly, there are growing reports of biliary infection in immunocompetent individuals as well, including cases of acute and chronic cholecystitis [18–20].

*C. belli* infections have equally been seen in patients following solid organ transplant [21–24]. More recently, there was a case of *C. belli* infection reported in a patient who underwent a small bowel transplant [25]. In these cases, the patients were successfully treated, without further sequelae and had no reports of extraintestinal manifestations. However, given the need for chronic immunosuppression use in these patients, reactivation remains a concern [26]. Also, it is not clear whether *C. belli* infections in these solid organ transplant recipients were derived from the donor or contracted independently by the recipient. There has been one previous report incidentally found *C. belli* in donor gallbladders, during a retrospective pathologic review of gallbladders [27]. Unfortunately, no further information was available on the recipients or donors in these cases.

## 2. Case Report

### 2.1. Recipient

The patient is a 59-year-old male who had struggled with oxalate nephrolithiasis since the age of 13, without formal workup. He previously underwent multiple lithotripsies, as well as a partial nephrectomy and remained relatively controlled with a baseline creatinine of 1.2-1.3 mg/dL (reference range: 0.70–1.30 mg/dL). Unfortunately, in September 2016, the patient progressed to chronic kidney disease, after an episode of dehydration. He was seen in our institution in November 2016 after presenting with an episode of acute on chronic renal failure. He had no renal reserve and was initiated on hemodialysis. Further history revealed a daughter with oxalate stones disease as well, raising concern for hereditary oxalosis; other serological studies were negative, and biopsy confirmed acute tubular necrosis (ATN) with oxalate nephropathy. Genetic testing was pursued, and the results showed an AGXT mutation consistent with a type 1 primary hyperoxaluria. All preoperative liver testing results were within the normal limits. Given this diagnosis, the patient was evaluated by the transplant committee, and a combined liver-kidney transplant was recommended [28, 29]. The patient underwent a combined orthotopic liver (OLT)-kidney transplant in July 2017. A donor cholecystectomy was done as per the standard protocol. Pathologic examination revealed *Cystoisospora belli* organisms. The patient was treated with trimethoprim/sulfamethoxazole (TMP/SMX) DS 800–160 mg every 6 hours for ten days followed twice daily for three weeks. There is currently no evidence of *C. belli* reactivation.

### 2.2. Donor

The donor was a 20-year-old Caucasian male who suffered an anoxic brain injury. He had no history of biliary disease/symptoms and had no evidence of acute or chronic cholecystitis, biliary disease, or other biliary disease at the time of donation. There was no reported history of acute or chronic diarrhea, and he was otherwise immunocompetent. He had no medical comorbidities, no prior surgeries, no history IV drug use, or other risky behaviors. He had no history of recent travel outside of the United States. Notable pretransplantation labs included bilirubin of 0.5, AST 62, ALT 76, and alkaline phosphatase of 49.

### 2.3. Pathologic Review

**Figure 1:**
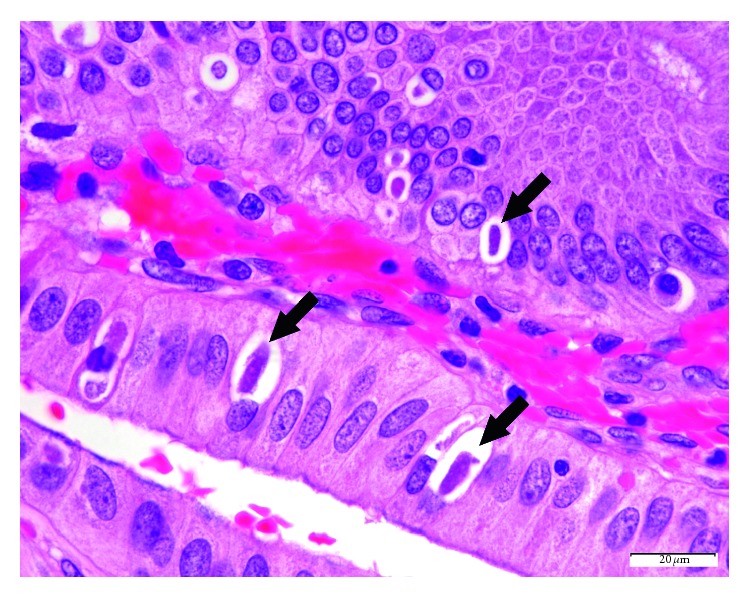
Donor gallbladder, H&E, 1000x. The characteristic morphology of *Cystoisospori belli* includes its banana-shape and perinuclear parasitophorous vacuoles (arrows) within the gallbladder epithelium, as seen here.

**Figure 2:**
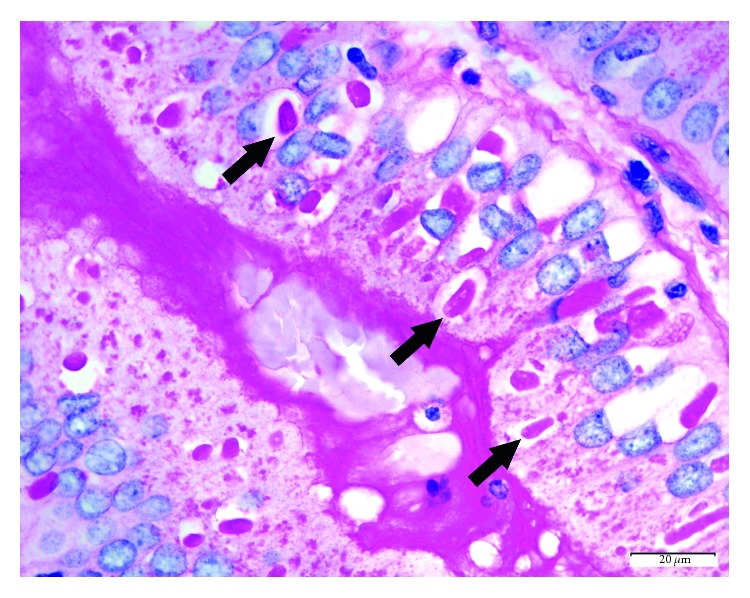
Donor gallbladder, periodic acid–Schiff stain with diastase (PAS/d), 1000x. Although not required for diagnosis, the parasites can be highlighted by a PAS/d special stain (arrows).

**Figure 3:**
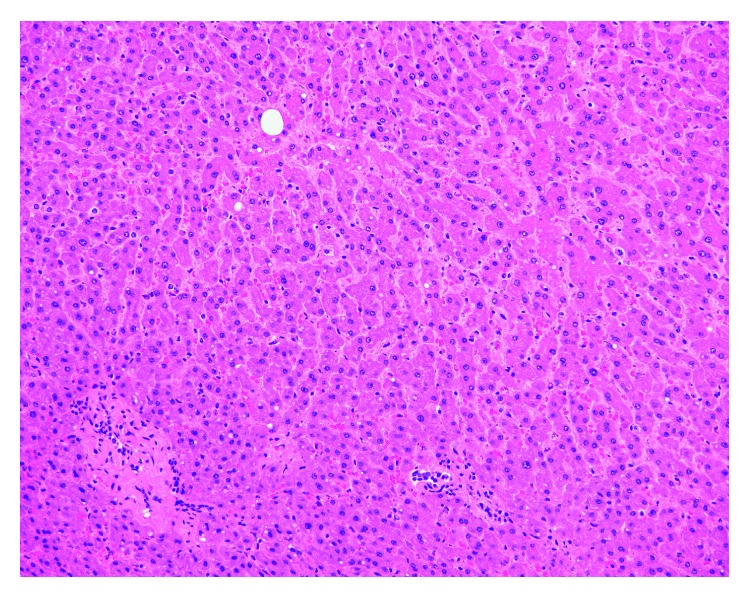
Native liver explant, H&E, 20x. Sections show an unremarkable liver with no significant fat, fibrosis, or inflammation.

The donor gallbladder specimen measured 5.6 × 2.1 × 0.6 cm. It had a tan-gray and smooth serosa, with a limited amount of attached adipose tissue. There were no pericystic lymph nodes, and the cystic duct was not obstructed. The lumen had no calculi and no erosion, and mucosa was tan-brown, with an average wall thickness of 0.3 cm. This was consistent with a grossly normal gallbladder. On H&E staining, oval-shaped intracellular structures, measuring approximately 20 *µ*m, were identified within the cytoplasm of the biliary epithelium, consistent with *C. belli*. (Figure 1) The background gallbladder was otherwise unremarkable [27, 30, 31]. The organisms were highlighted by the PAS/D special stain. (Figure 2). The liver histologic evaluation was otherwise unremarkable, with no significant fat, fibrosis, or inflammation. (Figure 3).

## 3. Discussion

*C. belli* infection is a rare cause of diarrhea, even in immunocompromised individuals. This is likely due to the rarity of the organism especially in the West, as well as improvement in prophylaxis in transplant patients and severely immunocompromised HIV patients. However, there is increasing awareness of the disease among practitioners [27]. There are multiple causes of infectious diarrhea in transplant patients including CMV, noroviruses, bacterial infections, and protozoans like cryptosporidium, Giardia, and toxoplasmosis [32–34]. In this case, the histologic diagnosis of *C. belli* was challenging because the background gallbladder was essentially unremarkable, requiring careful high-power examination to identify the parasite.

Luckily, liver transplant patients undergo a cholecystectomy at the time of transplant. However, in addition to the high rate of recurrent infection in immunocompromised (and some immunocompetent) patients [26, 35], *C. belli* infections are associated with extraintestinal dissemination and have been linked to arthritis [17], thrombotic thrombocytopenic purpura (TTP) and hemolytic uremic syndrome (HUS) [36]. In this case, the histologic diagnosis of *C. belli* was challenging because the background gallbladder was mostly unremarkable, requiring careful high-power examination to identify the parasite. The typical features described in the literature include epithelial disarray and vacuolated epithelium [14, 27], features not seen in all cases. However, if identified, the patient can be easily treated and given prophylaxis [37] against future reactivation. Additionally, if reactivation does occur, the clinician will have a higher index of suspicion than otherwise.

This case study equally highlights the ever-present concern of donor to recipient infection transmission in solid organ transplantation [38, 39]. The absence of specific protocols and guidelines to check for protozoal and intracellular infections, such as these, means that surgeons rely on the physician's index of suspicion to adequately screen for such organisms. Given the increasing use of high-risk donors [40–42] in organ transplantation, one must be aware of these below-the-radar type infections in order to decrease posttransplant complications. This case is important to raise awareness of *C. belli*, which is a challenging diagnosis to make and has only been rarely reported in the literature. To date, there have been two reports [21, 43] of *C. belli* infection after liver transplant. One occurred after eight months and the other after four years (with recurrence in two months). Although these were likely contracted through ingestion of contaminated water or food, reactivation from infected donor tissue is a possibility as this has not been previously recognized.

## 4. Conclusion

*C. belli* is an infectious protozoan, which rarely produces symptoms in immunocompetent individuals, but can cause severe, life-threatening diarrhea and dehydration, as well as extraintestinal symptoms in immunocompromised hosts. Posttransplant immunosuppression put liver transplant patients at risk of contracting the disease, not only through the usual routes but also through the process of organ transfer. Thus, a high index of suspicion is required when assessing donors and donor specimens to prevent the risks of transmission of this protozoan, as well as, other infectious agents during transplantation.

## Abbreviations

HIV/AIDS: Human immunodeficiency virus/acquired immunodeficiency syndrome
PCR: Polymerase chain reaction
ATN: Acute tubular necrosis
AGXT: Alanine-glyoxylate aminotransferase
OLT: Orthotopic liver transplantation
TMP/SMX DS: Trimethoprim/sulfamethoxazole double strength
AST: Aspartate aminotransferase
ALT: Alanine aminotransferase
H&E: Hematoxylin and eosin
PAS/D: Periodic acid–schiff–diastase
CMV: Cytomegalovirus
TTP: Thrombotic thrombocytopenic purpura
HUS: Hemolytic uremic syndrome.
